# Prevalence and predictors of overweight and obesity among women in the Gaza strip-Palestine: a cross-sectional study

**DOI:** 10.1186/s12889-020-08966-1

**Published:** 2020-06-05

**Authors:** Rima Rafiq El Kishawi, Kah Leng Soo, Yehia Awad Abed, Wan Abdul Manan Wan Muda

**Affiliations:** 1 School of Public Health, Al Quds University, Gaza City, Gaza Strip Palestine; 2 Program of Nutrition, School of Health Sciences, Health Campus, Universiti Sains Malaysia, 16150 Kubang Kerian, Kelantan Malaysia

**Keywords:** Body mass index, Obesity, Overweight, Prevalence, Associated factors

## Abstract

**Background:**

The prevalence of overweight and obesity among women of childbearing age is considered a public health concern. Few studies have been conducted in the Gaza Strip to determine the magnitude of overweight and obesity. This study aimed to determine the prevalence of overweight and obesity along with their associated factors among women in the Gaza Strip.

**Methods:**

A cross-sectional study was conducted to recruit a total of 357 mothers aged 18–50 years. Interviews were carried out among mothers to collect sociodemographic information, nutritional information, and physical activity. Anthropometric measurements [height, weight and waist circumference (WC)] were conducted with the mothers. Body Mass Index (BMI) was computed to determine the prevalence of overweight and obesity. Multinomial logistic regression was used to examine the associated factors of overweight and obesity.

**Results:**

The combined prevalence of overweight and obesity among mothers was (64.1%). The results of multinomial logistic regression showed the risk of overweight and obesity increased with age, the highest risk being in mothers aged > 33.0 years (OR = 2.7, 95% CI: (1.06,6.86)), and (OR = 5.72, 95% CI: (2.07,15.78)), respectively, compared to mothers aged < 33.0 years. Moreover, mothers with medium and high educational levels had a slightly higher risk of obesity (OR = 0.31, 95% CI: (0.15,0.64)), and (OR = 0.32, 95% CI: (0.12,0.82)) respectively than mothers with low educational level. Household income was positively associated with overweight and obesity. Mothers exposed to higher monthly income were more likely to be overweight or obese (OR = 2.64, 95% CI: (1.20, 5.83)), and (OR = 3.06, 95% CI: (1.28,7.29)), respectively. Nutrition knowledge was significantly associated with a high prevalence of obesity (OR = 1.20, 95% CI: (1.03,1.38)).

**Conclusions:**

This study showed a higher prevalence of overweight and obesity among Palestinian women than previous studies. Age, educational level, monthly income, and nutrition knowledge were associated with the prevalence of overweight and obesity, compared to other variables that were not associated with overweight and obesity such as location, work status, physical activity, and sitting hours. Urgent action is needed to tackle overweight and obesity among women. Effective intervention is required to increase nutrition knowledge among women to improve their eating behaviors.

## Background

Obesity is commonly defined as a condition of abnormal or excessive fat storage in the adipose tissue to the extent of health impairment [1]. Excess weight is considered a serious public health concern contributing to several preventable chronic diseases such as diabetes, hypertension, heart disease and stroke, different types of cancers, osteoarthritis, and reproductive conditions [2]. Body Mass Index (BMI) is the most widely used tool to measure overweight and obesity [1]. On the other hand, waist circumference (WC) is used as a convenient indicator of abdominal adiposity, and it is a better predictor of the risk of chronic diseases than BMI [3].

The prevalence of overweight and obesity among women of childbearing age is considered a public health concern. Maternal obesity can result in negative outcomes for both women and fetuses. The maternal risks during pregnancy include gestational diabetes mellitus (GDM), preeclampsia and caesarean delivery. While, the fetus is at risk for stillbirth and congenital anomalies [4]. Globally, the prevalence of obesity has reached a point at which 50.0% of women of childbearing age are either overweight or obese [5]. According to World Health Organization (WHO) in 2016, 39.0% of adults (≥ 18 years) were overweight and 13.0% were obese [6]. Obesity prevalence is high in both the high-income and low-to-middle-income countries, but the prevalence of obesity is expected to increase more rapidly in the latter [7]. The main factor contributing to overweight and obesity is the imbalance between energy intake and energy expenditure [8, 9]. Worldwide, people consume more quantities of foods with a high energy density and exhibiting low physical activity, so this could lead to overweight and obesity epidemics around the world [10, 11]. Several variables associated with obesity, including genetic and demographic variables, such as a family history of obesity, age, and sex, cannot be modified; however, obesity-associated lifestyle factors (diet and physical activity) are often modifiable [12]. Patterns of food intake and eating habits in the nearby Eastern Mediterranean Region have changed noticeably during the past four decades [13]. Available studies on Eastern Mediterranean countries showed that obesity has reached an alarming level in both children and adults [14]. During the last three decades, the prevalence of overweight and obesity has increased at an alarming rate in the Arab countries, and it is more noticeable in women [15]. Palestinian adults are not spared from overweight and obesity and the recent trends have shown that people at the middle-age are at the most risk of overweight and obesity especially among women [16]. The Palestinian community has been experiencing a nutrition transition, where studies have shown a high prevalence of nutrition-related chronic diseases such as heart diseases, diabetes, hypertension, and cancer, and their risk factors (lifestyle, and obesity) [17]. A study conducted in the Gaza Strip showed that the trends of overweight and obesity among Palestinian women in an urban area, a refugee camp, and a rural area were*:* 57.0, 66.8, and 67.5%, respectively [18]. In the Gaza Strip, few studies have been conducted to determine the magnitude of overweight and obesity. Therefore, the objective of this study was to determine the prevalence of overweight and obesity among women in the Gaza Strip and to examine their association with the independent factors.

## Methods

### Study design

A cross-sectional study was performed to determine the prevalence of overweight and obesity among women in the Gaza Strip along with their associated factors. Data were collected between April and October 2012. Face to face interviews and anthropometric measurements were carried out to eligible women of the selected households.

### Sample size

The sample size was calculated using the single proportion formula in the Epi-Info Software Revision (Version 2; 2002). Anticipated population prevalence of obesity (p) = 32.0%, which was among women in the Gaza Strip in 2008 [Al-Majdalawi J, Abed Y. Determinants of obesity among married women attended MCH clinics-Gaza Strip, unpublished Master thesis - Al Quds University], and level of significance = 5.0% (0.05). Therefore, the final number of *n* = 334. To account for an attrition rate 20.0%, the participants had to be recruited for this study as follows: 334 + (0.20 × 334) = 400 mothers.

A total of 400 households were selected and from each household a participant was recruited who met the inclusion and exclusion criteria of being a mother aged 18 to 50 years old and not pregnant.

Out of 400 households, 357 mothers were recruited with a response rate of 89.2%. Among the participants, 12 mothers refused to participate in the study, and we excluded 31 households who did not meet the inclusion criteria as 18 mothers were pregnant, and 13 households did not include mothers’ ages 18–50 years.

### Sampling method

A multi-stage cluster sampling process was used to find participants who met the inclusion and exclusion criteria of a mother aged 18 to 50 years who was not pregnant or lactating to avoid subject bias. The Gaza Strip is administratively divided into main five districts, of which three districts were selected: north of Gaza Strip, Gaza city, and south of the Gaza Strip [19].

Three clusters were chosen randomly from those three districts depending on the sociodemographic situation: Jabalia as refugee camp in the north of the Gaza Strip, and El Remal as an urban area in Gaza city, then Al Qarara as a rural area in the South of Gaza Strip. At the first stage, a number of areas were chosen randomly from the entire of each cluster, then, systematic random sample households were selected within each area in the urban, the refugee camp, and the rural respectively. The number of households selected from each cluster was weighted in proportion to the total population of women in childbearing age (19.1%). Hence, the distribution of households in each area was 220 from Jabalia camp, 140 from El Remal area, and 40 from Al Qarara (Table 1).

**Table 1:** Sample distribution

Area	Estimated population	Estimated mothers	Sample
Jabalia (Refugee Camp)	110,000	21,010	220
El Remal (Urban area)	70,000	13,370	140
Al Qarara (Rural area)	20,000	3820	40
Total	200,000	38,200	400

### Data collection

Data were collected using a structured questionnaire for face to face interviews. Validity of the questionnaire was checked and obtained by consulting a number of experts with experience and knowledge of the topic to make suggestions and judgment about the adequacy of the tools. Interviews were carried out among mothers to collect information on a number of predictors which could potentially affect the nutritional status of women such as sociodemographic information, nutrition information, and physical activity. The variables which are included in this study are: Socioeconomic and demographic data as follow: Area of residence was divided into urban, rural and refugee camp. Household monthly income and it was expressed in New Israel Shekel (NIS) as NIS 3.9 was equal to US$1. Household income was categorized into three classes (< 1000 NIS, 1000–2000 NIS and > 2000 NIS). Food or financial assistance was received from non-government agencies like UNRWA (United Nations Relief and Works Agency). Mothers were asked their age at their most recent birthday and it was categorized as (≤26, 27–33, > 33) years. Education level was defined as low level (elementary or below and preparatory), medium level (secondary); and high level (diploma/university level, and postgraduate). The mothers were asked about family size (number of family members), and the number of pregnancies.

The International Physical Activity Questionnaire (IPAQ) has been utilized internationally to obtain comparable estimates of physical activity patterns [20]. To measure the physical activity pattern of mothers, the researchers used the validated Arabic short-format version of the IPAQ. The questions were about the time mothers spent being physically active in the last 7 days, including questions about activities mothers did at work, as part of household chores to get from place to place, and in their spare time for recreation, exercise, or sport. In answering the questions, vigorous physical activities referred to activities that required vigorous physical effort and made mothers breathe much harder than normal. In contrast, moderate physical activities referred to activities that required moderate physical effort and made mothers breathe relatively harder than normal. Data collected with IPAQ can be reported as a continuous score. An average metabolic equivalent of task (MET) score was derived for each type of activity. In this study, sitting time is used as an independent proxy measure of sedentary behavior [21]. This measure included time spent sitting (in days and minutes/day) at work, at home, while doing coursework, and during leisure time (sitting at a desk, visiting a friend, reading, and watching TV). The nutrition knowledge of a participant was assessed using an adapted questionnaire from the National Coordinating Committee for Food and Nutrition, Ministry of Health, Malaysia, 1997. The questionnaire consisted of 16 questions included 5 categories addressing: food nutrients sources, different types of healthy food, healthy food and body functions, body weight and exercise, and food and its related to diseases. The median of the nutritional knowledge scores =13, it was used to separate the higher half of mothers’ scores distribution ≥13, from the lower half < 13. Validity of the questionnaire was checked and obtained by consulting a number of experts in health and nutrition from Al-Quds University, and the United Nations Children’s Fund (UNICEF) in the Gaza Strip. In addition, *Kuder–Richardson Formula 20* (KR-20) was calculated for each item and the general reliability for all items was 0.76. It was considered acceptable, as the acceptable value was from 0.70 to 0.95.

Pilot study questionnaires were distributed among 30 women before conducting the study, and after obtaining the results, the necessary changes were made. Anthropometric measurements were taken by two trained research assistants. The heights and weights of mothers were measured using the standard recommended procedures of the WHO [22]. SECA digital weighing scale (to the nearest 0.1 kg) and SECA body meter (with a precision of 0.1 cm) were used to measure the heights and the weights of the mothers, respectively, and mothers wore lightweight clothes and no shoes. The Body Mass Index (BMI) was computed with the following formula: weight (kg)/height (m)^2^. According to WHO, an adult BMI of less than 18.5 was considered to be under-weight, between 18.5 and 24.9 was normal, and between 25.0 and 29.9 was overweight. An adult with a BMI of 30.0 or higher was considered obese [1]. The waist measurement was obtained with a tape measure to the nearest 0.1 cm, with mothers wearing only light clothing around the waist area in a horizontal plane, midway between the inferior margin of the ribs and the superior border of the iliac crest. The WHO recommends sex-specific WC cut-off points for women (≥80 cm), thus, women with WCs of > 88 cm, face a high risk of cardiovascular disease (CVD) [23].

### Data analysis

Analyses were performed with the statistical software package version (SPSS) 22.0. Descriptive statistics such as frequency and proportion were used to describe the sample and to determine the prevalence of overweight and obesity among women in the Gaza Strip. Cross tabulations and Pearson’s Chi-square test were used to obtain the associations and strength of relationship between the independent and the dependent variables. Multinomial logistic regression analysis was performed to control for confounding factors and to determine the Odds Ratios (ORs). The univariate analysis was carried out to evaluate each independent variable for its unadjusted association with overweight or obesity. In the second, bivariate associations, we included all independent variables with *p* < 0.25 significantly associated with overweight or obesity, because they were considered important to evaluate factors associated with overweight or obesity. In the final model, the significance level was *p <* 0.05.

### Ethical issues

Ethical approval was obtained from the Helsinki Committee for Research Ethics in the Ministry of Health in the Gaza Strip, and ethical clearance was approved from University Sains Malaysia (USM) ethical committee. The written consent form was obtained from each mother before the interview. Moreover, mothers were informed that the questionnaires could be answered voluntarily, and that the information would be treated confidentially.

## Results

Out of 400 households, 357 were identified and screened to find mothers who met the inclusion and exclusion criteria and agreed to participate in this study giving a response rate of 89.2%.

Table 2 presents the mothers’ anthropometric characteristics. The mean ± SD weight and height of the mothers were 70.95 ± 15.46 kg and 1.59 ± 0.05 m, respectively. The mean ± SD WC was 91.12 ± 12.76 cm and the mean ± SD BMI was 27.90 ± 5.91 kg/m^2^. The results revealed that the combined prevalence of overweight and obesity was 64.1%. The prevalence of overweight/obesity was 34.5 and 29.6% respectively. Table 3 shows the prevalence of overweight and obesity increased as age increased, with the highest prevalence 33.3, and 47.7% respectively in the age > 33-years, (*p* < 0.001). The results showed that weight increased as income increased, the prevalence of overweight and obesity was significantly higher in participants with high family income 38.0 and 33.8% respectively, compared to those with low family income, (*p* = 0.022). Moreover, the prevalence of overweight and obesity was high 35.3 and 41.9% among mothers with high number of pregnancies, (*p* = 0.001). A multinomial logistic regression model was used to assess the odds for the sociodemographic and other variables to a participant being overweight and obese (Table 4). Our results revealed that as the age of mothers increased especially > 33 years, the risk of being overweight and obese increased (OR = 2.70; 95% CI: 1.06–6.86; *p* = 0.038) and (OR = 5.72, 95%: 2.07, 15.78; *p* = 0.001), respectively. In addition, higher educated mothers had a slightly higher risk of obesity than mothers with lower educational levels (OR = 0.32; 95% CI: 0.12–0.82; *p* = 0.017). The results revealed that mothers lived in households with higher monthly income (> 2000 NIS) or (1000-2000NIS) appeared to have significantly higher risks of being overweight (OR = 2.64; 95%CI: 1.20–5.83; *p* = 0.015) or (OR = 2.58; 95% CI: 1.40–4.74; *p* = 0.002) than those with lower monthly income (<1000NIS). Moreover, our findings further revealed that mothers lived in households with higher *household income* (>2000NIS) or (1000-2000NIS) were *significantly* associated with higher rates of obesity (OR = 3.06; 95% CI: 1.28–7.29; *p* = 0.011), or (OR = 2.36; 95% CIs: 1.21–4.62; *p* = 0.012), respectively than those with lower monthly income (<1000NIS). In addition, our data (Table 4) showed that as the scores of nutrition knowledge increased to a participant, the risk of being obese increased (OR = 1.20; 95% CI: 1.03–1.38.0; *p* = 0.016).

**Table 2:** Mothers’ Anthropometric characteristics (*n* = 357)

Variables	Frequency(n)	Percent (%)	Mean (SD)
Mother’s body weight (kg)			70.95 (15.46)
Mother’s body height (m)			1.59 (0.05)
Waist circumference (cm)^a^			91.12 (12.76)
< 80.0	62	17.4	
≥80.0	295	82.6	
Mother’s BMI			27.9 (5.91)
Underweight < 18.5	7	2.0	
Normal weight 18.0–24.9	121	33.9	
Overweight 25.0–29.9	123	34.5	
Obese class I 30.0–34.9	60	16.2	
Obese class II 35.0–39.9	34	9.5	
Obese class III ≥40.0	12	3.9	

^a^Ref. [23]

**Table 3:** Baseline characteristics of mothers in the Gaza Strip by BMI category (n = 357)

Variables	Normal weight (***n*** = 128)	Overweight (***n*** = 123)	Obesity (***n*** = 106)	***P***-Value (X^**2**^)
**Geographic location**				0.107
Urban	43 (43.0)	26 (26.0)	31 (31.0)	
Rural	13 (32.5)	19 (47.5)	8 (20.0)	
Refugee camp	72 (33.2)	78 (35.9)	67 (30.9)	
**Age (year)**				< 0.001 (42.73)*
≤ 26	57 (55.9)	31 (30.4)	14 (13.7)	
27–33	50 (34.7)	55 (38.2)	39 (27.1)	
> 33	21 (19.0)	37 (33.3)	53 (47.7)	
**Educational level**				0.017 (12.01)*
High	29 (36.7)	32 (40.5)	18 (22.8)	
Medium	60 (42.9)	46 (32.8)	34 (24.3)	
Low	39 (28.3)	45 (32.6)	54 (39.1)	
**Work status**				0.294
Housewife	122 (36.0)	114 (33.6)	103 (30.4)	
Employee	6 (33.3)	9 (50.0)	3 (16.7)	
**Household member**				0.090
3–6	92 (49.5)	61 (32.8)	33 (17.7)	
> 6	36 (21.1)	62 (36.3)	73 (42.6)	
**Household assistance**				0.492
Yes	63 (34.1)	62 (33.5)	60 (32.4)	
No	65 (37.8)	61 (35.5)	46 (26.7)	
**Monthly income (NIS)**				0.022 (11.49)*
> 2000	20 (28.2)	27 (38.0)	24 (33.8)	
1000–2000	43 (29.7)	58 (40.0)	44 (30.3)	
< 1000	65 (46.0)	38 (27.0)	38 (27.0)	
**Nutrition knowledge score**				0.078
< 13	63 (38.4)	62 (37.8)	39 (23.8)	
≥ 13	65 (33.7)	61 (31.6)	67 (34.7)	
**Type of physical activity**				0.203
Low	23 (29.9)	25 (32.5)	29 (37.6)	
Moderate	105 (37.5)	98 (35.0)	77 (27.5)	
**Sitting time (hour/day)**				
1–3	94 (37.5)	84 (33.4)	73 (29.1)	0.623
> 3	34 (32.1)	39 (36.8)	33 (31.1)	
**Number of pregnancies**				< 0.001 (30.88)*
1–5	90 (47.4)	64 (33.7)	36 (18.9)	
> 5	38 (22.8)	59 (35.3)	70 (41.9)	

Chi square test. *Statistically significant *P*-value < 0.05

1$ = 3.9NIS

High Educational level: Diploma, university level, and master’s degree/PhD; Medium Educational level: secondary; Low Educational level: elementary or below and preparatory

**Table 4:** Factors associated with body mass index (overweight and obesity) based on a multinomial analysis

Variables	Overweight (***n*** = 123)		Obesity (***n*** = 106)			
	OR	(95%CI)	***P***-value	OR	(95.0% CI)	***P***-value
**Age (year)**						
≤ 26	1	–	–	1	–	–
27–33	1.80	(0.91, 3.56)	0.092	2.21	(0.97,5.02)	0.057
> 33	2.70	(1.06, 6.86)	0.038*	5.72	(2.07,15.78)	0.001*
**Educational level**						
Low	1	–	–	1	–	–
Medium	0.56	(0.29,1.08)	0.086	0.31	(0.15,0.64)	0.001*
High	0.80	(0.35,1.83)	0.600	0.32	(0.12,0.82)	0.017*
**Monthly income NIS (1$ = 3.9NIS)**						
< 1000	1	–	–	1	–	–
1000–2000	2.58	(1.40, 4.74)	0.002*	2.36	(1.21,4.60)	0.012*
> 2000	2.64	(1.20, 5.83)	0.015*	3.06	(1.28,7.29)	0.011*
**Nutrition knowledge**	1.10	(0.95, 1.22)	0.243	1.20	(1.03,1.38)	0.016*

Adjusted weight gain for Overweight (BMI 25.0–29.9 kg/m2) or Obesity (BMI ≥30.0 kg/m2) vs. healthy weight (reference) associated with Sociodemographic and

Lifestyle-related variables. *Statistically significant (*p* < 0.05)

High Educational level: Diploma, university level, and master’s degree/PhD; Medium Educational level: secondary; Low Educational Level: elementary or below and preparatory

## Discussion

The combined prevalence of overweight and obesity among women aged 18 to 50 years is high (64.1%). While a previous study reported that 71.3% of Palestinian women aged 30 to 65 years were overweight and obese [24]. The differences in results can be attributed to variation in age groups. In the developing countries, obesity is not only a condition of high socioeconomic status (SES) groups, but the risks of obesity tend to shift to the lower SES groups towards the poorer groups [25, 26]. Our results revealed that the prevalence of obesity is 29.6%, which is a high trend associates with a high risk of non-communicable diseases. Palestinian women were observed to be particularly vulnerable to obesity-related diseases along with co-morbidities like diabetes [17]. In addition, the common abnormality in our participants was the majority of women had abnormal WC, which is a convenient indicator of abdominal obesity and it is related to cardiovascular risk factors [2]. Based on the multinomial logistic regression analysis, overweight and obesity increased as age increased. Our results revealed that the highest prevalence of overweight and obesity was seen among women in the age group (> 33 years). These findings are consistent with other studies reported that the prevalence of obesity increased with age [27, 28]. The association between excess weight and age can be explained by the physiological and behavioral changes within women that occur as a result of aging like the lowering in energy requirement at rest with aging [29, 30]. This generally indicates that women at an older age with decreasing physical activity help accumulate more weight [28]. Education was a typical indicator of SES associated with excess weight in developed countries, while income was in the developing countries [31]. The results of this study revealed that women with higher educational level had a slightly higher risk of being obese than women with lower educational level. Similar results reported that individuals with higher education levels were more likely to be overweight [28, 32]. Higher education helps women live a life that includes less physical activity and helps access energy-dense food which is considered to cause overweight or obesity [28]. While in other studies, results revealed that high prevalence of obesity was found among people who were less educated. As it is known that people with higher levels of education are healthier than people with lower levels of education [33]. Education is associated with awareness and attitude attainment that allows people to integrate healthy behaviors into their lifestyle [26]. Interestingly, in this study, nutrition knowledge was not a protective factor for obesity, it was found in the multinomial analysis that those with high nutrition knowledge scores were at high risk to be obese than those with low nutrition knowledge scores. That could be due to such knowledge wasn’t translated into good practices. Nutritional knowledge is positive when it’s translated to practice and results in better nutrition; otherwise, it is considered deceased/less knowledge [34]. A previous study was conducted in the United Kingdom showed that, despite a high level of awareness on healthy eating and many public health campaigns to encourage people to adopt healthy diets, obesity prevalence still increased [35]. Our results further revealed that the household’s income was positively associated with overweight and obesity. This study showed that women from non-poor households have significantly increased the risk of overweight and obesity than those belonging to poor families. It was suggested that high economic status was significantly associated with weight gain. The same results of other studies showed that a significant positive relationship between income and a high BMI [28, 36]. That could be explained by high calories food intake, with a lack of awareness of the health risk associated obesity, as well as a more sedentary lifestyle which is considered to cause weight gain [37].

However, our results revealed that all women were low or moderately active, but there was no significant association between physical activity and excess weight. [Abdeen et al. (2012)] recognized in his study low physical activity among Palestinian obese women, and that could be due to the limited availability of exercising facilities for Palestinian girls and women [16]. A similar result has been found in Arab countries, specifically, the prevalence of a sedentary lifestyle increased along with the nutritional transition, especially with the advancements in technology and transportation [13]. Women in most Arab states generally faced more barriers to the practice of physical activity than men because of social traditions and safety reasons. In Bahrain, 67.0% of women perceived that they had limited opportunities to participate in physical activity because most exercises and sports facilities are only provided for men [38]. This *study* has some *limitations* which have to be pointed out. The study design was cross-sectional to identify associated factors with overweight and obesity, which could not determine the cause and effect relationship. Case-control studies should be conducted in the future to address the risk factors of weight gain. In this study, we assessed physical activity as a subjective tool (IPAQ), instead of a more objective tool such as an accelerometer or pedometer.

## Conclusions

Our findings revealed a higher prevalence of overweight and obesity among Palestinian women than previously reported in other local studies. Therefore, obesity is a major public health problem among women in the Gaza Strip. Age, educational level, monthly income, and nutritional knowledge were associated with overweight and obesity among women in the Gaza Strip. There is a need for urgent action to develop health strategies to combat the growth of overweight and obesity. In addition, an effective nutrition education program should be implemented to help women to convey their nutrition knowledge into healthy dietary practices.

## Data Availability

Data are available from the authors upon reasonable request.

## Abbreviations

BMI: Body mass index
CI: Confidence interval
CVD: Cardiovascular disease
IPAQ: International physical activity questionnaire
OR: Odds ratio
MET: Metabolic equivalent of task
SES: Socioeconomic status
SPSS: Statistical package for social science
SD: Standard deviation
UNCIF: United Nations children’s fund
WC: Waist circumference
WHO: World health organization
